# Effects of Arts and Psychomotor Therapies in Personality Disorders. Developing a Treatment Guideline Based on a Systematic Review Using GRADE

**DOI:** 10.3389/fpsyt.2022.878866

**Published:** 2022-06-16

**Authors:** Suzanne Haeyen

**Affiliations:** ^1^Department ’Scelta', Expert Center for Personality Disorders, GGNet Centre of Mental Health, Apeldoorn, Netherlands; ^2^Special Research Group ’Arts and Psychomotor Therapies in Personality Disorders', HAN University of Applied Sciences, Nijmegen, Netherlands

**Keywords:** personality disorders, art therapy, music therapy, drama therapy, dance therapy, psychomotor therapy, systematic review, grade

## Abstract

What is the effect of arts and psychomotor therapies, using art, dance, drama, music, movement and body awareness, in personality disorder treatment? This was explored by developing a treatment guideline based on a systematic review using the GRADE system within the context of the Dutch national multidisciplinary guidelines for treatment of personality disorders. Conclusions were formulated by a work group and based on the scientific substantiation and were integrated with other indications in the functioning of arts and psychomotor therapies in personality disorders. The first general search yielded 1,900 records which was brought back to 53 full-texts. Ultimately, 1 RCT and 2 pilot studies were included. Recommendations for treatment are that arts and psychomotor therapies are included in treatment, independent of age, sex or specific diagnostic characteristics. Arts and psychomotor therapies can be considered for purposes of coming into emotional contact with difficult aspects of patients and their experiences, to work on goals such as regulation of emotions, stress, identity/self-image, self-expression, mood/anxiety, relaxation, changing patterns and social functioning. Enlisting arts and psychomotor therapies for patients with a personality disorder is recommended because they value these therapies and perceive these to be effective. It could be considered to ask arts and psychomotor therapies to provide a contribution to the diagnostic process, to the problem analysis *via* observation and to determining treatment indication and treatment goals. More research is needed.

## Introduction

This article offers an overview of the scientific evidence for arts or psychomotor therapy in treating personality disorders at present, based on an intensive literature study that took place within the context of the national multidisciplinary guideline for the treatment of personality disorders. It describes conclusions, further indications and recommendations for the use of arts and psychomotor therapies.

A personality disorder is a very common psychiatric disorder. At least one personality disorder occurs in 13.5 percent of the general population. The percentage for psychiatric patients and for addicts who have been treated is 60 and 56 percent, respectively (1). In present practice, arts and psychomotor therapies are often part of a psychotherapeutic or social psychiatric treatment for people with a personality disorder. These therapies are provided to individuals and to groups, customized or modular. Arts and psychomotor therapies are generally part of a treatment programme in a policlinic, day clinic or clinic. They are often embedded in multidisciplinary programmes in a consistent and shared therapeutic context, such as Dialectic Behavioral Therapy (DBT), Schema-focused Therapy (SFT) and Mentalization-based Treatment (MBT), psychodynamic psychotherapy or in general or generic psychotherapy treatments such as Acceptance and Commitment Therapy (ACT) or, for example, Guideline-Informed Treatment for Personality Disorders (GIT-PD). Arts and psychomotor therapy can also form part of the psychotherapeutic treatment of independent psychologists and psychotherapists in the context of interdisciplinary collaboration and professional networking. Arts and psychomotor therapies are used widely across the globe although the contexts differ a lot. There are multiple associations for arts and psychomotor therapies in Europe (e.g., EFAT, BAAT, FVB, ATI, NIGAT), North-America (AATA), South-America (CAT), Canada (CATA, AATQ, BCAT), Australia (ANZACATA), in the middle east (ICET), Asia (HKAAT, ATAS) and internationally (ATWB, IEATA).

Just like all other disciplines in healthcare, arts and psychomotor therapists must handle the demand for substantiation of their interventions. What evidence is available for the interventions and how do you determine what is good evidence?

Multidisciplinary guidelines are often developed at a national level for the treatment of people in a particular diagnostic category. This often takes place in an overarching institute or framework such as a national institute for health and care excellence or in this case, the Federation of Medical Specialists in association with the Trimbos Institute (1). For example, there is also a guideline for the multidisciplinary treatment of personality disorders. One guideline focuses on what, according to the present standards and criteria, is the best form or type of care for patients with a personality disorder. The guideline includes a large number of topics such as: the patient and family perspective in relation to treatment, diagnosis and needs assessment, psychotherapeutic interventions, nursing care and arts and psychomotor therapy, but also crisis intervention, pharmacotherapy, cost effectiveness and organization of care. A guideline is meant for all healthcare providers who are involved in the care of patients with a personality disorder. Evidence-based medicine (EBM) refers to the application of the best available research on clinical care, and what is required in order to integrate evidence with clinical expertise and patient values (2, 3). The object of EBM is to support the patient by contextualizing the evidence with their preferences, concerns and expectations. This results in a process of shared decision-making, where the values, circumstances and setting of the patient determine the best care. The widespread use of EBM in many healthcare disciplines (e.g., nursing, psychology, arts and psychomotor therapies, medicine) reflects the broad impact. EBM plays a prominent role in policy development: study data and informative decision making such as a declaration of legitimacy (4).

The primary question in this study was: What is the effect of arts and psychomotor therapies as treatment of a personality disorder? In answering this question, we make as great a distinction as possible between the various arts and psychomotor therapies, which include art therapy, dance, drama, music, movement and body awareness therapy. Arts and psychomotor therapies cover the following: arts therapy, drama therapy, dance therapy, music therapy and PMT. Play therapy is also reckoned to the arts and psychomotor therapies, but in this guideline it is omitted from consideration because this form of therapy is not used and has not been studied in relation to personality disorders. Arts and psychomotor therapies have an experiential, action-directed and creative quality and make methodical and targeted use of a wide range of working methods, materials, instruments and attributes, for example, with precisely a great deal of structure, or precisely very little. Feelings, thoughts and behavioral patterns that come forward *via* design, play, bodily sensations or movement, provide leads to awareness and introspection. This takes place by means of observation and *via* contact with others, regulation of impluses and emotions, by addressing patterns in feelings, thoughts, acting and practicing with new roles and skills (5–9, 90).

## Methods

This study concerned a systematic review using GRADE (Grading of Recommendations, Assessment, Development and Evaluations), a transparent framework for developing and presenting summaries of evidence and provides a systematic approach for making clinical practice recommendations (10–12). It is the most widely adopted tool for grading the quality of evidence and for making recommendations. The guideline work group consisted of representatives of each professional discipline (e.g., psychotherapists, psychologists, pharmacologists, nurses, trainers, different therapists) in mental health care practice as well as family and representatives. This group worked together and gave eachother feedback during the whole process. The process starts with the authors deciding what the clinical question is, including the population that the question applies to (13) for the systematic review—providing the best estimate of the effect size for each outcome, in absolute terms (e.g., a risk difference) (12). The authors rate the quality of evidence for each outcome. The quality of evidence often varies between outcomes (14). An overall GRADE quality rating can be applied to a body of evidence across outcomes, usually by taking the lowest quality of evidence from all of the outcomes that are critical to decision making (15). The work group followed the process of the present study critically in great detail and conclusions and recommendations were formulated and approved by the work group.

### Grade Format

The guideline texts used to answer the primary questions are drawn up in accordance with a set structure based on the GRADE method. The primary question for every form of therapy is: “is treatment X recommended for disorder Y?” This question is introduced for each therapy form using a brief introduction with a description of the nature of the treatment and the assumed mechanism, supported by references to the literature. This introduction is followed by the summary of scientific support, consisting of the discussion of the research assessed. This leads to results in a number of conclusions that are related to the primary question. It is followed by the transition from conclusions to recommendations. Four significant factors have been taken into account (16): (1) The consideration between favorable and unfavorable effects of the treatment; (2) The extent of certainty as to the estimated effects (uncertain proof of low quality, fairly certain proof of high quality); (3) The extent of certainty as to the values and preferences of patients (ideally, on the basis of systematically collected information but otherwise, at the estimation of the work group); (4) The workload entailed by recommending a treatment using the available means.

### Rating the Evidence

Depending on these factors, a treatment will be recommended or not recommended. A distinction is made between weak and strong reommendations. For a strong recommendation, one would recommend treatment X for all patients with Y. For a weak recommendation, this depends, for example, on the preferences of the patient involved. In this section, it is important to mention explicitly in this section on what basis a treatment is or is not recommended and also why the recommendation needs to be weak or strong. This is in fact the essence of Grade (working toward explicit and transparent descriptions of choices). This guideline working group opted for a strong recommendation if the advantages were greater than the disadvantages for almost all patients.

GRADE has four levels of evidence—also known as certainty in evidence or quality of evidence: very low, low, moderate, and high (Table 1). Evidence from randomized controlled trials starts at high quality and, because of residual confounding, evidence that includes observational data starts at low quality. The certainty in the evidence is increased or decreased for several reasons, described in more detail below (17).

**Table 1:** GRADE certainty ratings.

**Certainty**	**Relevance/Meaning**	**Preferred formulation**
Strongly in favor	The advantages are greater than the disadvantages for nearly all patients. All or almost all informed patients are very likely to choose this option. The authors have great deal of confidence that the true effect is similar to the estimated effect.	We recommend [intervention].
Weakly in favor	The advantages are greater than the disadvantages for a majority of the patients, but not for everyone. The majority of informed patients will probably choose this this option. The authors believe that the true effect is probably close to the estimated effect.	Consider [intervention], discuss the advantages and disadvantages.
Weakly against	The disadvantages are greater than the advantages for a majority of patients, but not for everyone. The majority of informed patients will probably not choose this option. The true effect might be markedly different from the estimated effect.	Be reticent about [intervention], discuss both advantages and disadvantages.
Strongly against	The disadvantages are greater than the advantages for nearly all patients. All or nearly all informed patients will probably not choose this option. The true effect is probably markedly different from the estimated effect	We do not recommend [intervention].

*Guyatt et al. (10)*.

### GRADE Is Subjective

There is by necessity a considerable amount of subjectivity in each decision. GRADE provides a reproducible and transparent framework for grading certainty in evidence (18). Evidence becomes less certain with each of the following: risk of bias, imprecision, inconsistency, indirectness, and publication bias. Authors have the option of decreasing their level of certainty by one or two levels (e.g., from high to moderate). GRADE is used to rate the body of evidence at the outcome level rather than the study level. The risk of bias can be rated using available tools (19). Certainty in a body of evidence is highest when there are several studies that show consistent effects. Evidence is most certain when studies directly compare the interventions of interest in the population of interest and report the outcome(s) critical for decision-making. Certainty can be rated down if the patients studied are different from those for whom the recommendation applies. Full information about GRADE can be found in the GRADE guidelines series (e.g., Guyatt et al., 2011).

A guideline text does not only include the result of systematic literature review but also includes for a large part the patient and family perspective in relation to treatment, diagnosis and needs assessment, psychotherapeutic interventions, nursing care and arts and psychomotor therapy, but also crisis intervention, pharmacotherapy, cost effectiveness and organization of care. A guideline presents the state of the art evidence as well as all other relevant aspects that should be taken into account in making clinical choices in treatment.

### Search Strategy/Review Protocol

In September 2019 a first general search was conducted of systematic reviews, meta-analyses and randomized controlled trials (RCTs) relating to personality disorders in Medline, Embase, PsycInfo, and the Cochrane Database of Systematic Reviews. The search strategy used can be requested (Ed.). In order to answer the aforementioned primary question focused on arts and psychomotor therapies these records were screened using the PICO (Table 2). In June 2020 also a supplementary search was conducted with the same search terms focused on arts and psychomotor therapies.

**Table 2:** PICO for search.

**P**	**People with a DSM classification for a personality disorder**
I	One of the arts and psychomotor therapy interventions: a. Arts therapies b. Drama therapy/psychodrama c. Dance/movement therapy d. Music therapy e. Psychomotor therapy
C	Treatment as usual (TAU) Standard treatment Waiting list condition or no treatment Comparison with one or more other treatment(s)^a^ ^a^Note: No conclusions can be drawn from this comparison that apply to the effectiveness of an individual treatment
O	Crucial: - Quality of life/positive mental health - Important: - Emotional functioning - Suffering and malfunctioning - Symptomatic recovery - Social recovery - Failure (substitute for adverse events)

## Results

### Included and Excluded Studies

**Figure 1:**
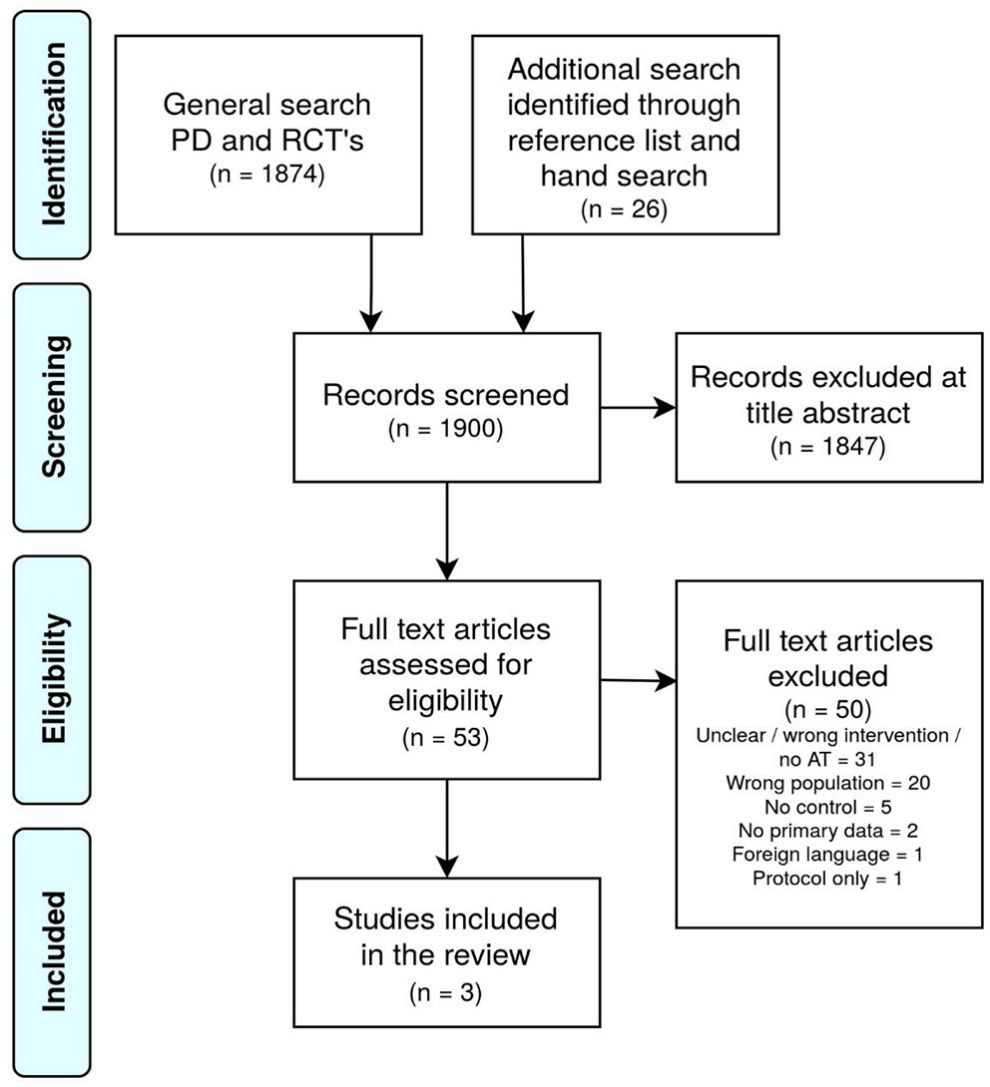
Prisma diagram of the search results.

The first broad search (see Figure 1) yielded 1,874 results. The supplementary search yielded 26 articles. From these results (*n* = 1,900) 53 were selected for full-text inspection. The majority of all articles found (*n* = 31) were excluded because the intervention was unclear or did not involve arts and psychomotor therapies. Twenty articles were excluded because the research took place *not only* among patients with personality disorders, and this group was *not analyzed separately*. Furthermore, it proved that there was no control group in a number of studies, or that no effect data were available for a different reason (*n* = 7). In addition, reasons for exclusion were absence of a control group (*n* = 5), no primary data (*n* = 2), foreign language or protocol only (both *n* = 1). Ultimately, 1 RCT and 2 pilot studies were included to answer the primary question (Tables 3, 4).

**Table 3:** Outcome measures.

**Outcome measure of protocol**	**Outcome measure of study**
Quality of life/positive mental health	Wellbeing and psychological flexibility
Emotional functioning	General mental functioning (subscale emotional functioning) Emotional state (mode)
Symptomatic recovery	General psychological complaints (social functioning, emotional functioning, social role)
Social recovery	Subscale social functioning

**Table 4:** Characteristics of the studies included.

**Study**	***N***	**Country**	**Therapy duration**	**Follow-up**	**Average age [SD]**	**Female (%)**	**Outcomes measured**
Haeyen (20, 21)	57	Netherlands	10 weeks group sessions	5 weeks	37.48 (SD = 10.45)	70.25%	Psychological flexibility General psychological functioning (social/emotional and in societal role) Emotional mode
Broek (22)	10	Netherlands	4 individual sessions (from 3 months of treatment)	–	40.7 (SD = 7.4)	0	Emotional mode: healthy modes
Keulen-de Vos (23)	9	Netherlands	5 individual sessions	–	38.2 (SD = 7.6)	0	Emotional modes: vulnerability

### Scientific Substantiation

#### Haeyen et al. (20, 21)

In a recent RCT (20) of the effect of art therapy, 57 patients with a personality disorder cluster B/C were randomized between a weekly art therapy group (1 1/2 h, 10 weeks) and a waiting list group. After a follow-up of 5 weeks, the primary outcome, psychological flexibility (decrease in avoidance of experience, more acceptance of unpleasant inner experiences), measured with the Acceptance and Action Questionnaire-II. Effect sizes were calculated using the change in Cohen's d, an indication of the effect over time. This brought the AAQ-II-total to a small effect [Δd = 0.11, *d* post-test −0.44 (−0.97, 08) 95% CI], whereas large effects were found on mental functioning, measured using the Outcome Questionnaire 45 (OQ45-total score/decrease in complaints: Δ*d* = −1.67 *d* post-test −1.24 (−1.81, −0.68) 95% CI). The experimental group showed a decline in personality disorders pathology in the degree of presence of maladaptive schema modes and measures using the Schema Mode Inventory (SMI) [less impulsiveness Δ*d* = −1.66, *d* post-test −088 (−1.42, −0.33) 95% CI, detachment Δ *d* = −1.31, *d* post-test −1.04 (−1.59, −0.48) 95% CI, vulnerability Δ*d* = −1.24, *d* post-test −0.64 (−1.18, −0.11) 95% CI, and punitive behavior; Δ*d* = −1.29, *d* post-test −0.88 (−1.43, −0.34) 95% CI]. The scores on the adaptive schema modes as perceived from and through pleasant feelings, spontaneity (happy child mode) Δ*d* = 1.55, *d* post-test 1.19 (0.63, 1.75) 95% CI, and self-regulation (healthy adult mode) Δ*d* = 1.60, *d* post-test 1.38 (0.80, 1.96) 95% CI, showed an increase. Art therapy decreased not only personality disorder-pathology and maladaptive modes, but also helped patients to develop adaptive positive modes that indicate positive mental health and self-regulation.

In a second study that reports on the same RCT (21), additional data was analyzed to investigate whether arts therapy was effective in increasing mental wellbeing (positive mental health) or on decreasing psychological complaints (mental illness), or both. Five questionnaires [AAQ-II, Dutch Mental Health Continuum-Short Form (MHC-SF), Mindful Attention Awareness Scale (MAAS), SMI-II adaptive—maladaptive scales and two subscales of the OQ45] were divided into two domains: positive mental health and mental illness, in order to compare the effect on these two domains. The effect of art therapy on the indicators for positive mental health was between Δd = 0.52 on the MHC-SF (social wellbeing), *F*_(2, 30)_ = 28.05, *p* < 0.01, and Δd = 1.46 on the AAQ-II, *F*_(2, 30)_ = 60.00, *p* < 0.01. The results also showed large effect sizes for outcomes for mental illness [Δd = −0.82 on the OQ45 Interpersonal relationships scale, *F*_(2, 30)_ = 27.83, *p* < 0.01 and Δd = −1.32 for the SMI maladaptive modes, *F*_(2, 30)_ = 109.85, *p* < −0.32]. The average effect on the indicators for positive mental health was Δ*d*= 1.06 and on indicators for mental illness Δ*d* = −1.09. Art therapy proved to be not only a generic intervention to improve wellbeing and quality of life, but also a specific therapy that reduced specific symptoms of the psychiatric disorder.

#### Van den Broek et al. (22)

In a pilot RCT (22) the effectiveness of art, drama and psychomotor therapy was studied by evoking various emotional schema modes in forensic patients. Ten male patients with cluster B personality disorders were allocated on a random basis in a clinical trial of Schema Focused Therapy (SFT) vs. “normal” forensic treatment [Treatment As Usual (TAU)]. The effects of arts therapy vs. verbal psychotherapy and of SFT vs. TAU on emotional modes were studied. Patients showed significantly more healthy emotional modes in the arts therapy (*d* = 0.80) than in the verbal psychotherapy (*T* = 7.00; *p* < 0.05). Art therapy, drama and psychomotor therapy and SFT proved to have the potential to call up healthy emotional expressions in forensic patients with a personality disorder.

#### Keulen-De Vos et al. (23)

In another pilot study, evoking emotions by means of drama therapy was studied in male delinquents with a cluster B personality disorder (23). Nine male patients in a forensic psychiatric clinic followed a protocol of 5 drama therapy sessions. Emotions were tested using the Mode Observation Scale (MOS) before and after each session. Participants showed significantly more emotional vulnerability in all experimental intervention sessions; After session 2, the Vulnerable Child mode was seen more often (M = 1.88, SE = 0.28) compared to the baseline scores [*M* = 1.0, SE = 0.007, *t*_(7)_ = −3.13, *p* = 0.017, *d* = 1.18]. This also proved to be the case in session 3 [*M* = 2.06, *SE* = 0.30 after the session compared with *M* = 1.09, *SE* = 0.06 before the session, *t*_(7)_ = 3.26, *p* = 0.014, *d* = 1.23]. In contrast to this, they were compared to before the session [*M* = 1.00, *SE* = 0.008, *t*_(8)_ = 1.41, *p* = 0.19, *d* = 0.50]. It is apparent from the results that drama therapy offers opportunities in calling up emotional vulnerability in forensic patients with a cluster B personality disorder. However, there are limitations to this study: it is not discussed what is done with the emotions after they are evoked in drama therapy. The purpose of evoking emotional vulnerability in forensic clients remains somewhat unclear.

## Conclusions

**Table:** 

**⊕⊕⊖⊖**	*Outcome measure: improving psychological flexibility, positive mental health and self-regulation* There are indications that art therapy has a favorable effect on patients with a personality disorder. # Haeyen (20, 21)

*# downgraded for risk of bias and for imprecision*.

**Table:** 

**⊕⊕○○**	*Outcome measure*: *decreasing personality pathology and maladaptive functioning (decreasing vulnerability)* There are indications that arts therapy has a favorable effect on patients with a personality disorder. # Haeyen (20, 21)

*# downgraded for risk of bias and for imprecision*.

**Table:** 

**⊕○○○**	*Outcome measure: evoking emotions* It is not certain, but there are indications that patients with a cluster B personality disorder show more emotional vulnerability in drama therapy. # van den Broek (22, 23)

*# downgraded for imprecision (2x) and for risk of bias*.

## From Evidence to Recommendations

### Quality of Evidence

The scientific evidence was formed by merely one RCT and 2 pilot RCTs, which can be explained by the fact that research culture is still rather limited in this field.

- The effect sizes of the change in the outcomes in the RCT of Haeyen et al. (20, 21) indicate improvement in the experimental group after the intervention. The imprecision on account of the limited size of the study (*N* = 57) is contradicted by an adequate sample size calculation. The drop-out analysis shows that no bias occurred due to drop-out. On account of a few methodological restrictions (blinding the allocation), we are cautious as to the robustness of the scientific evidence.
- The pilot RCTs of van den Broek et al. (22) and Keulen-de Vos et al. (23) are very small (*N* = 10/*N* = 9). This greatly limits the quality of evidence.

### Other Indications in the Functioning of Arts and Psychomotor Therapies in Personality Disorders

There are also other indications that arts and psychomotor therapies are effective in the treatment of personality disorders. These indications were found in the other relevant studies that were excluded for the scientific substantiation-underpinning in this document. In view of the availability of scientific evidence, they are still relevant for mention here. However, these are presented here with mainly qualitative descriptions because they serve a different goal than the studies included for scientific substantiation. This concerns, for example, RCTs in which not the entire population, but only part of the group studied had a personality disorder, open studies with a pre- post-design or qualitative research. The group of people with a personality disorder were not analyzed separately in these studies, and so they were excluded as scientific evidence. To give an impression of the clinically most relevant studies, they are briefly described below. These studies may be relevant because a substantial part of the group studied had been diagnosed with a personality disorder. The final recommendations should be seen as an outcome of the complete process and as a guide line for daily practice in treatment of PD patients.

#### Art Therapy

In 319 patients, Karterud and Pedersen (24) studied the effect of the components of a group-focused, brief day treatment for personality disorders. Eighty-six percent of the patients had a personality disorder, which generally concerned an avoidant personality disorder or borderline personality disorder. The treatment effect was evaluated with the question: How much profit did you gain from the following groups during treatment? The profit from the art therapy scored significantly higher (*p* < 0.001) than all other groups. This score was set off against the outcomes on (*inter alia*) Global Assessment of Functioning (GAF) and the Group Style Instrument (GSI). The score of the art therapy group correlated significantly (*p* = 0.005) with the “overall profit” gained from the programme. The multiple regression analysis indicated the presence of a stronger effect in the arts therapy group. The authors point out that patients with a personality disorder greatly appreciate art therapy, particularly on account of the “as if-situation” (25), that offers a safe method by which to explore and express their experiences and to assign, or mentalise, them by means of their own objects in the form of work papers. In an RCT by Green et al. (26), half of 28 chronically psychiatric 'outpatients', including patients with a personality disorder, were randomly assigned to a supportive art therapy group supplementing TAU, and half received TAU. The results of this study show that the patients in the experimental group showed improvement in social functioning, their attitude toward themselves, and in their own self-confidence.

#### Drama Therapy

Kipper and Ritchie (27) conducted a meta-analysis on the basis of 25 experimental studies of psychodrama techniques such as role switching, twinning and role-playing om various target groups, including those with personality disorders. Outcome measures of diminishing stress and avoidance, self-esteem, conflict management, reality check, empathy and positive self-image are mentioned. They concluded that these techniques contribute positively to the development of empathy, to coming in contact with their own subjective world and being able to view situations (e.g., *distancing*).

In the field of psychotherapy, experiential work has also been viewed as helpful. For example, Popolo et al. (28) conducted a small randomized clinical trial (N = 40) of metacognitive interpersonal therapy–group (MIT-G) vs. treatment as usual (TAU). In MIT-G, role-playing techniques (drama techniques) were used for patients with personality disorders as to gain awareness in their patterns and drives when interacting with others. In this study patients in the MIT-G arm reported significant improvements on symptoms, functioning, interpersonal problems and changes in depression and anxiety of medium magnitude, and large changes for alexithymia. It is stated that in group psychotherapy experiential techniques are useful to practice experiences in a controlled and safe environment and to actually feel these experiences in the body (29).

#### Music Therapy

Schmidt (30) studied the effects of 2 months of music therapy in a group (1½ h twice a week) of 34 patients with a borderline personality disorder (BPS) and 29 patients with “general neurotic/psychosomatic problems” (54% of total N had the diagnosis BPS). The most important descriptive results show that, after the study, patients met een BPS were satisfied about music therapy, reported better perception, felt themselves more able to enter into new contacts, and felt calmer and more relaxed. Gold et al. (31–33) demonstrated that music therapy seemed to be an effective addition for patients with a low level of motivation (*N* = 144, only 6 of which had a BPS). Research by Gebhardt et al. (34) showed that patients with personality disorders (*n* = 34, 18.8% of *N* = 610) more often use music to reduce negative thoughts and to achieve relaxation than do 'healthy' test subjects. In an RCT, Musical Attention Control Training (MACT) for psychiatric patients with psychotic characteristics, a number of which also had a comorbid personality disorder (*N* = 35, 7 of which had a PD), MACT seemed to be effective in facilitating attention skills (35). A randomized pilot study (*N* = 10) shows that group music therapy for LVB patients (IQ 70–85) with characteristics/features of a cluster B personality disorder seemed to be effective in improving regulation of emotions and mitigating avoidance and passive coping (36).

#### Dance and Movement Therapy

In a parallel trial, Leirvag et al. (37) compared the treatment effects of psychodynamic group therapy (PGT) and body awareness group therapy (BAGT) with policlinical day care for female patients with serious personality disorders (*N* = 50). Patients who followed BAGT showed significantly higher scores in the field of general and interpersonal functioning. Over time, they reported more satisfaction with the therapy and the group climate. A body awareness intervention (Basic Body Awareness Therapy; BAT) was studied in an RCT by Gyllensten et al. (38). This concerned psychiatric patients (*N* = 77, only 7 of which (18%) with a PS). BAT showed improvements on psychiatric symptoms, body posture and movement, self-efficacy, sleep and physical coping. The effect of short-term dance and movement therapy on depressive and anxiety disorder symptoms in patients with a personality disorder (*N* = 20) were studied by Punkanen et al. (39). Measurements before and after the therapy sessions showed a significant decrease in depressive symptoms and better recognition of their own feelings. A Systematic Review focused on Dance Movement Therapy (DMT) for personality disorders yielded the inclusion of four articles with opinions of experts (40). Six overarching themes were found for DMT interventions for PD: self-regulation, interpersonal relationships, integration of self, coping experiences, cognition and expression and symbolisation in movement/dance. Results of a meta-analysis based on 33 studies (*N* = 1,078) by Koch et al. (41) among patients with a wide range of psychiatric problems suggest that Dance Movement Therapy and dance are effective for increasing quality of life and decreasing clinical symptoms such as depression and anxiety. Positive effects were also found in the increase of subjective wellbeing, positive mood/feelings, influence, and body image.

#### Psychomotor Therapy

In a pilot study by Zwets et al. (42), the effect of psychomotor therapy (PMT) as a supplement to Aggression Replacement Therapy (ART) was studied in the treatment of aggressive gedrag. Most patients in this study (*N* = 37) had a personality disorder (anti-social, narcisisstic PD and PD NAO). Clinically significant improvements were observeerd of aggressive behavior, social behavior and self-reported anger, but there were no significant differences in treatment effects on these primary outcomes. A small improvement was found on secondary outcomes such as body awareness during anger and coping behavior in the experimental group, with PMT compared to the control group. A study by Hutchinson et al. (43), a quasi-experimental setup with 37 psychiatric patients, 33% of whom had a personality disorder, showed that increasing physical fitness by means of a structured practice programme (15–20 weeks) can have a favorable effect on mood, psychological wellbeing, self-image, self-esteem and leads to less depression, anxiety and stress. Research by Knapen et al. [(44, 45), *N* = 119] also gives instructions for the increase in physical fitness and the improvement of self-image of psychiatric patients, including patients with a personality disorder in applying targeted-specific, focused-targeted psychomotor therapy. Comparative research with before and after measurements shows that group therapy focused on bodily awareness and using experiential techniques is more effective than psychodynamic group therapy to reduce problematic functioning of serious personality disorders (37). In an overview article by Sanderlin (46) a number of studies were described of the treatment of excessive anger and aggression dysregulation in populations with an antisocial personality disorder. The article is about prisoners, juvenile delinquents and hospitalized adolescents with impulse control problems. It was shown in four RCTs that aggression regulation training, sometimes combined with relaxation training and social practical training (twee RCTs, *N* could not be accounted for), leads to a significant improvement in aggression regulation. The combination of cognitive therapy and relaxation training is thought to have the most impact. An elaboration of PMT in personality disorders can be found in Drewes et al. (47). A multi-center RCT is taking place to study the effectiveness of schema therapy for older persons with a personality disorder, enriched with PMT arrangements (48, 49). A number of relaxation methods are used in PMT, such as functional relaxation (50), progressive relaxation (51, 52), autogenic training of Schulz (53), training in breathing (54, 55). A pilot RCT for borderline personality disorders produced indications for a useful contribution of mindfulness exercises, also used for PMT (56). These methods might well be useful in learning to control the tension level and regulation of emotions by patients with a personality disorder. In practice, a number of modules have been developed for aggression and impulse regulation in which PMT- a and b offers are included [for example: (57, 58)]. There is often very little contact on the part of these patients with their own body awareness on account of chronic overstrain and for the patients themselves, increasing tension is not adequately observable. A pilot study with time series design for a mixed group of personality disorders and eating disorders showed that a PMT module of aggression regulation had an effect on coping with anger by patients who excessively internalize their anger (59).

### Contribution to Observation, Drawing Up Treatment Indications and Objectives

Arts and psychomotor therapists often provide an appreciated contribution in practice to observation, for purposes of diagnostics and drawing up treatment indications and goals. Observations of therapists can be of great importance to confirm a diagnosis of a personality disorder, and they can stipulate in greater detail what is of great importance to diagnose and confirm the health and care needs in more detail (60). They have also developed several instruments for this purpose.

Arts therapists, for example, regularly make use of the Diagnostic Drawing Series (DDS). This test, developed in the United States, is based on the DSM-5 and makes use of an objective structure analysis of three drawings (61). The DDS thus contributes to the diagnostics. The drawings are scored on structural attributes. The test must be conducted by an arts therapist who is well-trained in this. Mills (62) studied a group of 32 patients with a borderline personality disorder. The drawings were scored blind on forty image characteristics and then were compared with other diagnostic groups. This produced a profile with a statistically significant indication for the drawings of the borderline personality disorders with frequently occurring characteristics. In the drawing of a tree: disintegration and usage of much space (67–99%); in the third drawing (request for feeling): inclusion, color mixture (not in the first or the second) and abstraction. The study has a large-scale set-up and leaves little space for subjective interpretation. The instrument is described in an instruction manual (63). There is a clearly described control group. The DDS has high realiability among assessors; 84.2% of the items were scored correspondingly (Cohen's kappa 0.57) (64, 65). The DDS offers treatment indications because the test provides a profile of the patient relating to various appeals (dealing with structure, with appeal to expression and feeling and with task, motivation, willingness or the possibility to reflect, content of the information and the need to express oneself in this regard).

Drama therapists also make use of the Six Part Story Method (6PSM). The 6PSM is a projective technique in which a patient produces a fictitious story using structured instructions of the therapist. Research of Dent-Brown (66) and Dent-Brown and Wang (67) has shown that the level of pessimism and failure in a story of the patient's represents the seriousness of the borderline-personality disorder of the writer. 6PSM is said to be primarily of importance as a form of qualitative feedback to the patient.

Arts and psychomotor therapies are often combined with Schema Therapy. In the actual practice of treatment of personality disorders, it can be seen that arts therapy tallies closely with the combination of Schema Therapy and Positive Psychology (68, 69). Research makes use of the combination of arts therapy and Schema Therapy and endorses it [e.g., (22, 70)]. In a qualitative analysis by Brautigam (71) of the evaluation of a multidisciplinary clinical Schema Therapy treatment of patients with complex personality issues, arts therapies comes forward as a place for positive emotions and for practicing adaptive skills (healthy adult). The following quote from this study is about practicing this with adaptive skills:

“*Even so, it took me a great deal of effort, the fear of doing it wrong, to say the wrong thing, it has been my experience that this is not so simple, that you simply need to let go of control and improvise, and I can do that too.” (patient in drama therapy)*

In a year's research of multidisciplinary clinical Schema Therapy (including 3 different arts therapies) with positive psychological interventions, with patients with complex personality disorders, the average score of wellbeing was 1.34 (*SD* 67) on a scale of 5 at the start of treatment. A very low score in comparison with the Dutch population and also lower than the norm group of personality disorders. In the follow-up (6 months after the treatment) these patient scores were, on average, 2.33 (*SD* 1.12). That is still less than average compared to the Dutch population, but above average compared to the norm group of personality disorders. A large effect size came forward from this (72–74).

### General Instructions and Indications

In conclusion, it comes forward from the other relevant studies that arts and psychomotor therapies can be used, and are used often, for the purposes of coming into emotional contact with aspects of the surrounding world that are more difficult to reach, so as to be able to work on goals such as regulation of emotions, stress and tension, identity/self-image, self-expression, mood/anxiety, relaxation, changing patterns and social functioning. Psychomotor therapy is often cited as an option for improving physical fitness, body image, relaxation or the treatment of aggression and impulse regulation problems. Enlisting arts and psychomotor therapies for patients with a personality disorder is recommended because they value these therapies and perceive these to be effective (see also Patient perspectives).

The above described effects of arts and psychomotor therapies also come forward within the various panel discussions among arts therapists (75, 76). Specifically for young and for elderly people, despite the fact that there are hardly any studies of the effectiveness of arts and psychomotor therapies for young people or elderly people with personality pathology, in clinical practice, people see added value in the integration of arts therapy in a multidisciplinary treatment, primarily in the specialized mental healthcare institutions. There are indeed no reasons to assume that the recommendations as formulated for adults might not be applicable to young people as well as elderly people.

It can also be commented that a large number of studies of multidisciplinary treatment programmes comprise one or more forms of arts and psychomotor therapies. These programmes are found to be effective, but the influence on the effects found of the various aspects of treatment were not studied separately. For example, Styla (77) described positive results of treatment programmes for Personality Disorders in which psychodrama was part of the treatment offered. Van Dijk et al. (49) decribe the effect of a group-focused SFT programme enriched with PMT for the elderly. Many more examples can be cited [e.g., (78, 79)].

### Balance Between Desired and Undesired Effects

- No unwanted effects or negative side effects of arts and psychomotor therapies are known. It is necessary to mention that certain conditions have to be met like in any psychotherapeutic intervention such as safety, so that feedback can be processed in a regulated way [e.g., (80)]. A well-trained arts and psychomotor therapy is aware of the importance of this and will handle this as needed. However, safety and adverse effects need to be studies in future trials, though the observations made by this work group indicate that arts and psychomotor therapies are very likely safe.

### Patient Perspective

- The majority of patients with a personality disorder say that they value art therapy highly and perceive it to be effective [among others, (24, 81, 82)]. The importance of this is recognized in multidiciplinary collaboration and in independent practice. Arts and psychomotor therapies may be motivating and in a positive sense may contribute to the willingness to take part in treatment.
- In a longitudinal study by Haeyen et al. (81) among 528 patients with a personality disorder who received art therapy aimed at personality disorders, these patients report on repeated measurement times that they benefitted much and often from art therapy. This was not part of any RCT and was therefore excluded in this guideline as scientific evidence, and then primarily in the areas of emotional and social functioning. According to them, the five highest scoring objectives were: (1) expression of emotions, (2) improved (more stable/more positive) self-image, (3) making their own choices/autonomy, (4) recognition, insight into, and change of personal patterns of feelings, conduct and thoughts and (5) dealing with their own limitations and/or vulnerability. The scope of the observed profit went together with factors such as a non-judgmental attitude on the part of the therapist, feeling that he or she was taken seriously, and sufficient freedom of expression in order to experience in addition to the structure offered. Age, gender and diagnosis did not predict the scope of the experienced benefit. It was stipulated that arts therapy offers a broad target group as well as much profit, and so it can be used broadly. The benefit experienced and its increase over time showed a significant (Δ*R*^2^ = 0.03, *p* < 0.05) relationship with the extent to which patients perceived that they could give meaning to emotions in visual art.
- Which form of arts and psychomotor therapies is indicated differs per individual. This depends in part on whether the patient can and will open up: his or her affinity with arts therapy means and the possibilities experienced in the type of therapy concerned, and partly on specific diagnostic or personal characteristics. It is therefore important to give shape to this in consultation with the patient so that he or she can also take the lead in this regard and experience it.

### Perspective of Those Closely Involved

- Those who are closely involved find it important that enlisting arts and psychomotor therapies for clinical purposes in connection with effectively using treatment time is preferable over and above forms of worthwhile use of time and activities, so that it is possible to work on personal therapeutic goals as stated in the topicgroup for arts and psychomotor therapies of the guideline development group as well as in topic groups with other professionals (75, 76).

### Professional Perspective

- Arts and psychomotor therapies are often part of a psychotherapeutic or social-psychiatric treatment (60). Day clinical psychotherapy should be conducted by a multidisciplinary team, consisting of at least a (clinical) psychologist and/or psychotherapist, a psychiatrist, sociotherapist(s) and arts and psychomotor therapist(s). Part-time programmes and full-time programmes that do not work on the principles of (day) clinic psychotherapy often include arts and psychomotor therapies as well. Arts and psychomotor therapies are also very well possible in an independent setting in the context of interdisciplinary collaboration/professional networks. In this way, it is often part of a broader treatment programme. This makes it difficult to isolate its specific effect in research (83). Nevertheless, its value is broadly recognized by professionals specialized in the treatment of personality disorders, both nationally [e.g., (84)] and internationally [e.g., (85)].
- The combination of arts and psychomotor therapies with state-of-the-art psychotherapeutic treatment methods serve as a fruitful combination in practice; this is the general clinical experience (5, 70). Thanks to their experiential nature, encouraging mentalisation, arts and psychomotor therapies are often perceived as catalysts in this interaction or as catalysts of psychotherapy, as stated by a number of psychologist and psychotherapists (60).
- It is important that arts and psychomotor therapies are carried out by a qualified arts and psychomotor therapist with certified training for this purpose (5, 60).
- Arts and psychomotor therapists offer therapeutic group therapy which they independently stylise mostly without the presence of a co-therapist, as is the case in many psychotherapies. This entails extra responsibility in handling the therapy situation. It also asks for a good team collaboration with attention to safety and security, regular consultation as well as intervision and supervision (5).
- Optimally catering to the therapy can be encouraged by regularly evaluating the therapy together with the patient or patients (70).

### Budgetary Means

- Compared to the costs of specialized psychotherapies, arts and psychomotor therapies have a modest impact on budetary means. Moreover, arts and psychomotor therapies are used relatively often in a group context.

### Organization of Care

Many effective day treatment programmes comprise forms of arts and psychomotor therapies, individual and/or in groups (85–87). These therapies therefore are often available in practice.

- A number of psychotherapeutic treatment methods, such as the SFT and Emotion Regulation Training, for example, also make use of experiential treatment techniques. This is closely aligned with arts and psychomotor therapies, and these methods often work closely and directly with psychotherapists and with an arts/psychomotor therapist. Experts in various disciplines are of the opinion that arts and psychomotor therapies can be integrated in, and serve as an addition, for example, to dialectic behavioral therapy, SFT en MBT. This point of view comes forward in expert panels (75, 76) and is also evident from descriptive literature [e.g., (70, 88, 89)].

### Societal Perspective

- Deploying arts and psychomotor therapies at an early stage of treatment could contribute to willingness to be treated and patient motivation, an optimal link to the patient (personalized care) and an efficient course of treatment.

## Recommendations for Research

More substantial studies are needed to prove the effect of art therapies in PD research. It is recommended to make use of long term studies with a respectably sized sample.

Because arts and psychomotor therapies are mainly situated in multidisciplinary treatment settings, it can be hard to isolate their effects. However, effort should be made to isolate the causal effects of these therapies. This could be achieved by pre- and post-session measurements or by designs in which the arts/psychomotor therapy is the only intervention that makes the difference between groups (TAU vs. TAU plus arts/psychomotor therapy or arts/psychomotor therapy vs. waiting list/passive control). An other option is to add qualitative research to a study of a multidisciplinary program to investigate the contribution of each of the treatment elements.

## Recommendations for Practice

The recommendations about the use and effects of arts and psychomotor therapies in mental health care practice were based on the integration of the conclusions coming from the scientific evidence combined with all various other considerations and reflect the final opinion of the working group.

- It is recommended that a treatment programme for patients with personality disorders considers including the use of arts and psychomotor therapies.
- As part of treatment, offering art therapy or another form of arts and psychomotor therapies should be considered, independent of age, sex or specific diagnostic characteristics.
- It is recommended to educate patients about the various arts and psychomotor therapies and to include patient preferences in the process of together deciding on the indication for arts and psychomotor therapies.
- Psychotherapeutic verbal and arts and psychomotor therapies methods could be used within an ambulant, part-time, day-clinical or policlinical treatment, so as to ensure that patients with differing affinities, possibilities and learning styles are all able to respond and that the use of these techniques is tailored to the preferences of the individual and delivered upon negotiation with the patient.
- The use of arts and psychomotor therapies can be considered for purposes of coming into emotional contact with difficult aspects of patients and their experiences.
- It could be considered to ask arts and psychomotor therapies to provide a contribution to the diagnostic process, to the problem analysis *via* observation and to determining treatment indication and treatment goals.

## Data Availability Statement

The original contributions presented in the study are included in the article/supplementary material, further inquiries can be directed to the corresponding author/s.

## Author Contributions

The author confirms being the sole contributor of this work and has approved it for publication.

## Conflict of Interest

The author declares that the research was conducted in the absence of any commercial or financial relationships that could be construed as a potential conflict of interest.

## Publisher's Note

All claims expressed in this article are solely those of the authors and do not necessarily represent those of their affiliated organizations, or those of the publisher, the editors and the reviewers. Any product that may be evaluated in this article, or claim that may be made by its manufacturer, is not guaranteed or endorsed by the publisher.
